# Using a dynamic adherence Markov model to assess the efficiency of Respiratory Medication Therapy Adherence Clinic (RMTAC) on asthma patients in Malaysia

**DOI:** 10.1186/s12962-018-0156-1

**Published:** 2018-10-19

**Authors:** Yee Vern Yong, Asrul Akmal Shafie

**Affiliations:** 1 Formulary Management Branch, Pharmaceutical Services Programme, Ministry of Health Malaysia, Selangor, Malaysia; 2 Discipline of Social & Administrative Pharmacy, School of Pharmaceutical Sciences, Universiti Sains Malaysia, 11800 Penang, Malaysia

**Keywords:** Asthma, Adherence, Pharmacist, Cost-effectiveness

## Abstract

**Background:**

Respiratory Medication Therapy Adherence Clinic (RMTAC) is an initiative by the Ministry of Health (MOH) Malaysia to improve patients’ medication adherence, as an adjunct to the usual physician care (UC). This study aimed to evaluate the cost-effectiveness of combined strategy of RMTAC and UC (RMTAC + UC) vs. UC alone in asthma patients, from the MOH Malaysia perspective.

**Methods:**

A lifetime horizon dynamic adherence Markov model with monthly cycle was developed, for quality-adjusted life year (QALY) gained and hospitalization averted outcomes. Transition probabilities of composite asthma control and medication adherence, utilities, costs, and mortality rates due to all causes were measured from local data sources. Effectiveness, exacerbation rates, and asthma mortality rates were taken from non-local data sources. One-way sensitivity analysis (SA) was conducted for assessing parameter uncertainties, whereas probabilistic SA (PSA) was conducted on a different set of utilities and effectiveness data. Costs were adjusted to 2014 US dollars ($). Both costs and benefits were discounted at a 3% rate annually.

**Results:**

RMTAC + UC was found to be a dominant alternative compared to UC alone; $− 13,639.40 ($− 109,556.90 to $104,445.54) per QALY gained and $− 428.93 ($− 521.27 to ($− 328.69)) per hospitalization averted. These results were found to be robust against changes in all parameters except utilities in the one-way SA, and for both scenarios in PSA.

**Conclusions:**

RMTAC + UC is more effective and yet cheaper than UC alone, from the MOH perspective. For the benefit of both MOH and patients, RMTAC is thus recommended to be remained, and expanded to more healthcare settings where possible.

**Electronic supplementary material:**

The online version of this article (10.1186/s12962-018-0156-1) contains supplementary material, which is available to authorized users.

## Background

Asthma is a heterogeneous chronic respiratory disease that is usually characterized by chronic airway inflammation. Various pharmacological and non-pharmacological asthma managements have been developed to help patients achieve good symptom control and reduce future risk of exacerbation. Medication adherence is one of the most common factors of having poor asthma control [1]. In a survey across 14 countries, the median adherence rate for asthma patients is only 67% [2]. Non-adherence has been proven to worsen asthma control [3], increase risk of future exacerbation [4, 5], and increase asthma-related mortality rate [6]. These can adversely affect the society (patients themselves, healthcare payer, healthcare provider, employer, and other related parties) from both clinical and economics perspectives.

Acknowledging the importance of medication adherence, the Ministry of Health (MOH) Malaysia has initiated a pharmacist-managed Respiratory Medication Therapy Adherence Clinic (RMTAC) in public healthcare facilities. Targeted at non-adhered patients through individual-tailored non-pharmacological management, RMTAC is an adjunct to the usual care (UC) clinic, where physicians in specialist clinic see patients on routine appointments and focussed much lesser on non-pharmacological management. A comparison between RMTAC and UC is detailed in Table 1. Despite being initiated since year 2008, there is lack of information regarding its efficiency. Using modelling method, this study aimed to evaluate the cost-effectiveness of combined strategy of RMTAC and UC (RMTAC + UC) vs. UC alone in asthma patients, from the MOH Malaysia (major public healthcare provider and payer) perspective.

**Table 1:** Comparison between RMTAC and UC

Components	RMTAC	UC
Pharmacological management		•
Assess and monitor:		
Lung function	•	•
Asthma control	•	•
Medication adherence	•	•
Inhaler technique	•	•
Education on the disease and self-management	•	
Identification and education of individual asthma trigger factors	•	
Monitor and detect issues on the disease management, including pharmacological and others	•	

Components indicated for RMTAC and UC (represented as •) are formally structured as routine practice in the RMTAC protocol and physician clinic, respectively. The components for UC are not necessarily limited to those indicated in the table; others may just be non-routine practices

RMTAC, Respiratory Medication Therapy Adherence Clinic; UC, usual care

## Methods

### Model overview

The cohort in the model was 50-year old asthma patients with poorly controlled and/or low adherence with at least a prescribed inhaled corticosteroid and a short-acting beta agonist for the last 3 months, regardless of their phenotypes. The chosen starting age was based on the mean age of the sampled RMTAC cohort from a local public hospital. Those under RMTAC + UC were recruited into RMTAC during month 1, followed up 3 monthly till month 16, and thereafter biannually. The outcome measures were expressed in quality-adjusted life-years (QALYs) and number of hospitalizations. Costs and benefits were discounted at 3% per annum [8]. All costs were adjusted to 2014 US dollars ($1 = Malaysian Ringgit 3.22) [9, 10]. RMTAC was considered cost-effective if the incremental cost-effectiveness ratio (ICER) was below the Malaysian society threshold of $9,006 per QALY gained [7]. This study was approved by the Medical Research Ethics Committee of the Ministry of Health Malaysia (NMRR-12-372-11920).

### Model structure

It was decided to develop a new model although there were a few existing Markov models in the literature. This was because the existing models did not adequately consider medication adherence; adherence was commonly incorporated externally by examining different scenarios [11] or making assumptions [12, 13]. These approaches, however, lack the flexibility to directly capture adherence change frequently observed in practice. Consequently, there will be differences in the outcomes that can lead to different decision being made when the model resembles closer to real-world situation [14].

**Fig. 1:**
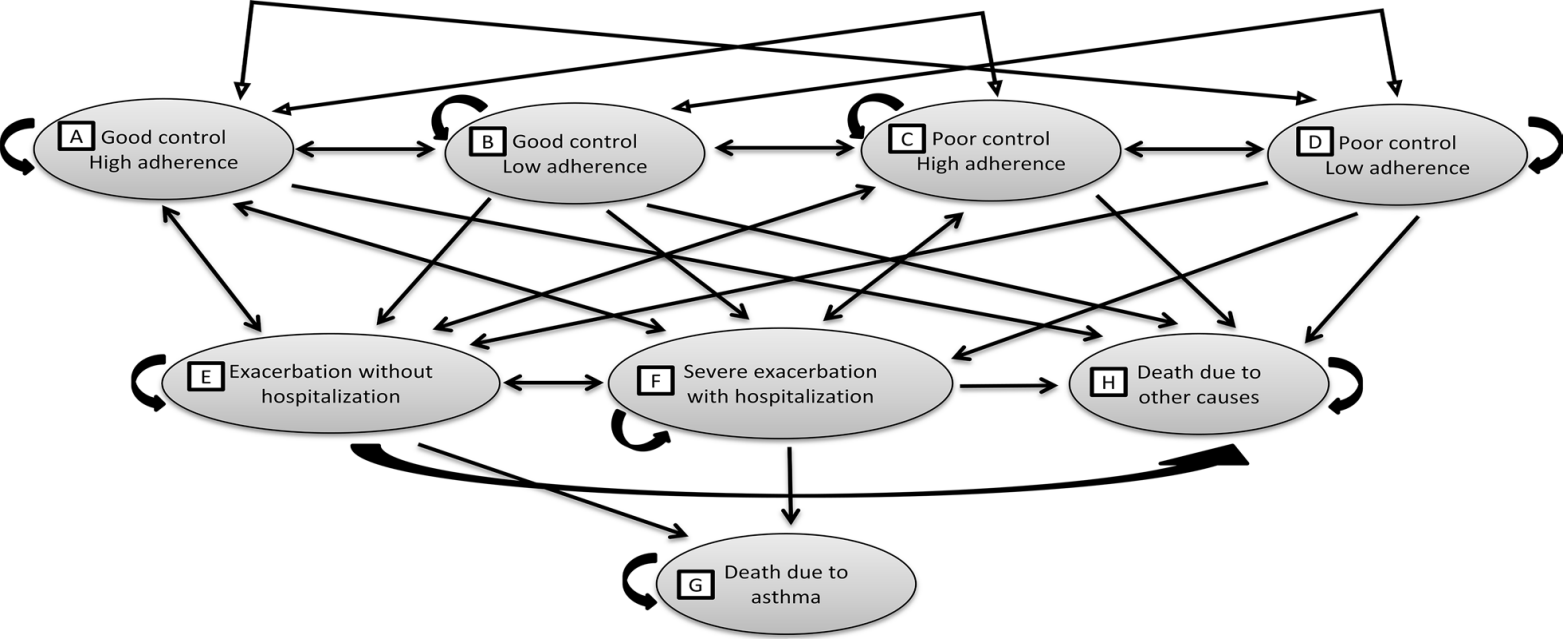
A dynamic adherence Markov cohort asthma model. The model was developed using Microsoft^®^ Excel 2007 (Microsoft Corporation, United States of America). The simulated cohort enters from either one of the three asthma control-adherence states (B, C, and D). Then after a cycle length of 1 month, they either transit to other health states or remain in the current state. There are two absorbing states here, death due to asthma and other causes. The one-way arrow indicates a single direction of transition from one state to the other, whilst the two-way arrow indicates that transition to and fro between two states is possible. The curved arrow indicates that the simulated cohort remains in the current state after a cycle

Therefore, a Markov cohort model that incorporated adherence dynamically was developed using Microsoft^®^ Excel 2007 (Microsoft Corporation, United States of America) (Fig. 1). This type of model was chosen because asthma is a chronic disease characterized by recurrence of events, and it can model future outcomes in a longer time horizon [15]. The differences in duration of acute asthma exacerbation and hospitalization do not make any real differences in transition probabilities; hence the cycle length is 1 month. As asthma is a lifelong disease, the model followed patients till they were dead or reached the age of 105 years.

There were eight health states in this model. Medication adherence was explicitly joined with the asthma control as a composite input of effectiveness. Medication adherence was not combined with the exacerbation states in this model because the latter was a direct effect of the combination between asthma control and medication adherence, which was the beginning state of the model. The hypothetical cohort began either from state B (good control-low adherence), C (poor control-high adherence), or D (poor control-low adherence). The cohort might die due to asthma (state G) when they transited into acute exacerbation [state E (urgent or emergency visit) or F (hospitalization)] from their current state. Although there were chances that they might die due to exacerbation before they could reach any healthcare facility on time, the risk is low and non-significant [16, 17].

This model has four assumptions. First, for simplicity sake, the model assumed that asthma exacerbations severity is mutually exclusive although it was possible for a patient to have more than one type of exacerbation in one cycle time. Secondly, although asthma control might not immediately improved to good level, the medication adherence level was assumed to be high once recovered from exacerbation especially if the exacerbation was triggered by external agents such as polluted air instead of medication non-adherence. Nevertheless, for exacerbation events due to poor medication adherence, the mean adherence level was good at 2 weeks after hospital discharge [18]. Third, the cohort’s pharmacological management and existing co-morbidities issues were assumed to be well managed and treated, which otherwise might pose a ‘false negative’ impact of an existing non-pharmacological intervention. Fourth, it was assumed that the RMTAC + UC cohort had RMTAC follow-up until they die.

### Parameter input

#### Transition probabilities

The transition probabilities between states A–D were calculated from RMTAC patient records of a local public hospital. Data on asthma control (assessed by Asthma Control Test^®^ (ACT)) and medication adherence (assessed by Modified Morisky Adherence Scale 8-Item) were collected from the records for this purpose. In this model, an ACT score of 19 and below defined poor asthma control, whereas an adherence score of below 6 defined low adherence level. The sample size was considered small relative to the true number of RMTAC records in that hospital over 8 years, mainly because two different versions of medication adherence tool had been used and their scores were not interchangeable; hence only RMTAC records that used the latest version of adherence tool were accounted for. Two methods were used to calculate the transition probabilities: (1) a simple and straightforward method for the observed 1-month transition probabilities (N = 16) and (2) a matrix algebra method for the others (N = 52) to predict 1-month transition probabilities as there were insufficient observed 1-month transition probabilities. For resulted value of zero even after both observed and predicted transition probabilities were combined, an addition of one to all observed transition counts as an uninformative prior (according to Bayesian theory) [19] was done prior to recalculating the probabilities for those transitions in that state.

Due to data feasibility issue, it was not possible to collect data for transition probabilities between states A–D under UC arm. Given that the effectiveness of RMTAC + UC vs. UC in improving medication adherence and/or asthma control was unknown, published literatures on similar pharmacist intervention were sought. There was no known literature that studied on both outcomes as a composite. Only one study that published effectiveness data on medication adherence, but as a secondary outcome [20]. Therefore, an effectiveness data on asthma control (as the primary outcome) from a study based on community pharmacists in Australia was employed; the odds of a pharmacist intervention that can result in good asthma control is three times more likely than without intervention (OR 3.06 (95% CI 1.63–5.73)) [21]. This data was first converted to relative risk [22] before applying to the respective transition probabilities calculated for RMTAC + UC arm to obtain the transition probabilities between states A–D under UC arm.

The probabilities of good and poor asthma control patients to get an exacerbation were obtained from an American observational study (N = 2942) [23], assuming that a zero score for Asthma Therapy Assessment Questionnaire meant good control whilst ≥ 1 score meant poor control, and that the adherence level in that study population was low. The probability calculated for state E was made up of that study’s data on both oral steroid bursts and Emergency Department (ED) visits, whereas that for state F was made up of data on hospitalizations. On the other hand, the risks of exacerbation in high adherence level patients stratified by asthma control level were obtained from an American observational study (N = 298) [5] and converted to probabilities [24], assuming that high adherence level was 76%–100% administration of prescribed inhaled corticosteroids and that risk referred to both types of exacerbation i.e. states E and F. The probabilities of achieving good and poor asthma control after an exacerbation were obtained from an American cross-sectional study (N = 2250) [25], assuming that 0–2 dispensing of short acting beta-agonist inhalers per year was good asthma control level, and that unscheduled visits meant both oral steroid bursts and ED visits. The probability of having an exacerbation after another was obtained from an observational study (UK population; N = 211,807) [26], assuming that it was equal for both states E and F. The probability of mortality following exacerbation in state E was obtained from a Spanish observational study by Morell et al. [27]. The age-stratified data on the mortality following exacerbation in state F was obtained from an American observational study (N = 65,831) [28]. The age-stratified data on the mortality due to all causes was obtained from the Malaysian Statistics Department [29]. Where appropriate, the monthly transition probabilities were calculated by using the formula *p* = 1 − e^−*rt*^ [30] where *p* is the monthly probability, *r* is the monthly rate, and *t* is the duration of 1 month.

Table 2 shows the transition probabilities as inputs of the model.

**Table 2:** Parameter inputs of the model

Parameter	Value	Range		Distribution
		Low	High	
Monthly transition probabilities between states A–D under RMTAC + UC				
A → B	0.122	0.03365	0.35673	Dirichlet
A → C	0.152	0.04763	0.39112	Dirichlet
A → D	0.030	0.00304	0.23896	Dirichlet
B → A	0.382	0.18072	0.63454	Dirichlet
B → C	0.032	0.00306	0.26248	Dirichlet
B → D	0.058	0.00895	0.29784	Dirichlet
C → A	0.184	0.08224	0.36201	Dirichlet
C → B	0.008	0.00042	0.13430	Dirichlet
C → D	0.043	0.00867	0.18760	Dirichlet
D → A	0.088	0.01812	0.33530	Dirichlet
D → B	0.051	0.00708	0.28827	Dirichlet
D → C	0.088	0.01812	0.33530	Dirichlet
Effectiveness factor				
Asthma control	OR 3.059	1.632	5.733	Log normal
Medication adherence	OR 1.89	1.08	3.30	Log normal
Monthly probabilities of low adherence level patient to have an exacerbation				
B → E	0.09273	0.07489	0.11021	Beta
B → F	0.00412	0.00330	0.00495	Beta
D → E	0.23237	0.19068	0.27191	Beta
D → F	0.05390	0.04335	0.06432	Beta
Risks of high adherence level patient to have an exacerbation				
A → E	HR 0.72	0.34	1.51	Log normal
A → F	HR 0.72	0.34	1.51	Log normal
C → E	HR 0.59	0.37	0.95	Log normal
C → F	HR 0.59	0.37	0.95	Log normal
Monthly probabilities of having good/poor asthma control after an exacerbation				
E → A	0.02528	0.01962	0.03132	Beta
E → C	0.10484	0.07127	0.16332	Beta
F → A	0.02394	0.01862	0.02961	Beta
F → C	0.10738	0.07261	0.16990	Beta
Monthly probabilities of having an exacerbation after a recent exacerbation				
E → F	0.047	0.0376	0.0564	Beta
F → E	0.047	0.0376	0.0564	Beta
Monthly probability of mortality after an exacerbation that does not involve hospitalization				
E → G	0.000059	0.000048	0.000071	Beta
Utilities input for base case analysis				
A	0.5583	0.4435	0.6731	Beta
B	0.5583	0.4435	0.6731	Beta
C	0.5316	0.3788	0.6844	Beta
D	0.5316	0.3788	0.6844	Beta
E	0.5311	0.4254	0.6368	Beta
F	0.3842	0.2882	0.4802	Beta
Utilities input for PSA				
A	0.5598	0.4588	0.6608	Beta
B	0.5598	0.4588	0.6608	Beta
C	0.5316	0.3788	0.6844	Beta
D	0.5316	0.3788	0.6844	Beta
E	0.4514	0.3589	0.5439	Beta
F	0.2919	0.2091	0.3747	Beta
Monthly direct cost ($)*				
A (maintenance)	38.78	33.77	43.78	Gamma
B (maintenance)	39.70	34.70	44.71	Gamma
C (maintenance)	42.48	37.46	47.50	Gamma
D (maintenance)	38.67	35.62	45.64	Gamma
E (ED management)	13.50	12.53	14.46	Log normal
F (hospitalization)	552.13	468.03	636.23	Gamma
RMTAC recruitment (cycle 0)	12.38	9.30	15.48	Gamma
RMTAC 3 monthly follow-up (cycle 1–15)	2.23	1.91	2.54	Gamma
RMTAC biannual follow-up (cycle 16 and above)	1.11	0.96	1.27	Gamma

A, good control–high adherence; B, good control–low adherence; C, poor control–high adherence; D, poor control–low adherence; E, exacerbation without hospitalization (Emergency Department visit); F, severe exacerbation with hospitalization; G, death due to asthma; PSA, probabilistic sensitivity analysis; RMTAC, Respiratory Medication Therapy Adherence Clinic; UC, usual care

* Monthly direct cost is expressed in 2014 US dollars ($)

The methods of calculating the transition probabilities are detailed in Additional files 1 and 2.

#### Utilities

The utilities for states A–F (Table 2) were obtained from a local study and measured using standard gamble method [31]. The utility for “well controlled asthma” and “poor controlled asthma” corresponded to states A–D, whereas utility for “severe exacerbation with urgent or ED visit”, and “severe exacerbation with hospitalization” corresponded to states E and F, respectively. The measured utilities here considered asthma control alone; although medication adherence is expected to affect the health-related quality of life, its association with the latter is inconsistent [32, 33, 34,35]. Hence, the utilities for states A and C were assumed to be the same for states B and D, respectively.

#### Costs

The unit costs of maintenance (states A–D), acute (state E), and hospitalization (state F) events were obtained from a local study and measured using activity-based micro-costing approach [36]. The unit costs of states A–D were prorated monthly according to the frequency of visits annually, as suggested by clinical experts; states A (6 monthly), B (4 monthly), C (2 monthly), and D (3 monthly) (Table 2). Using the same methods [36], the unit costs of RMTAC at recruitment and follow-up stages (Table 2) were measured. The resources involved were time spent by personnel, fixed assets, maintenance of the room, and consumables.

### Validation

The model was externally validated by comparing the life years in the simulated cohort with the national life expectancy of a 50 year old [37]. The life expectancy of both asthmatics and non-asthmatics were considered to be similar because the mortality due to all causes of asthma patients was found to have a non-significant age, smoking, social and medical confounders-adjusted hazard ratio when compared against non-asthmatics in a prospective study [38].

### Sensitivity analysis

One-way sensitivity analysis was performed on all parameters, within their 95% confidence intervals or an arbitrary range of ± 20% if confidence intervals were unavailable. The discount rate was also varied with 0% and 5%. The confidence intervals for the transition probabilities between states A–D were calculated using Wilson’s method [39].

Probabilistic sensitivity analysis (PSA) with 10,000 Monte Carlo simulations was performed to address three uncertainties. First was the parameter uncertainty on the base case. Second, the effectiveness data on asthma control in the base case was replaced with that on medication adherence; OR of 1.89 (95% CI 1.08–3.30) [20]. Third, the utilities in the base case were replaced with a different set of utilities measured from a bigger sample size; utilities were measured using the same methods [31] on another sample of subjects (N = 24) and combined with those used in the base case. However, repeated measures of the utility for poor asthma control (states C and D) was not possible due to illogical consistency findings in the study [31] which has yet to be further explored; hence, the utilities used in base case for these states remained in PSA. The inputs for the second and third PSA scenario are as in Table 2. The results of PSA were expressed in incremental cost-effectiveness ratio (ICER) (95% credible intervals (CI)).

## Results

### Base-case analysis

**Fig. 2:**
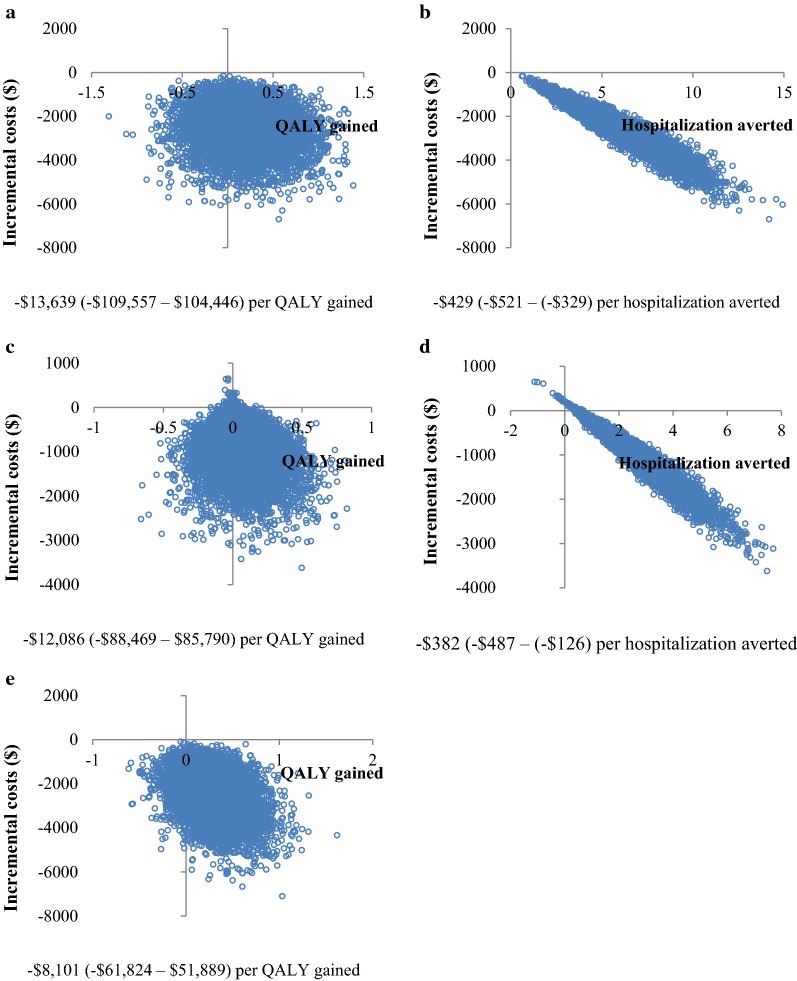
Results of probabilistic sensitivity analysis. This figure shows the results of Probabilistic Sensitivity Analysis on base-case (**a** and **b**), effectiveness factormedication adherence (**c** and **d**), and a different set of utilities (**e**). The results are expressed in incremental cost-effectiveness ratio (95% credible intervals).

The results for base-case analysis are presented in Table 3 and Fig. 2a, b. The number of life years was similar between RMTAC + UC and UC. It was also very close to the national life expectancy of a 50 year old i.e. 27.8 years [37], hence confirming the external validity of the model. With an incremental cost of $− 2409.86 ($− 4454.42 to ($− 841.22)), the mean number of QALY gained was 0.18 (− 0.45 to 0.83), whilst that of hospitalization averted was 5.62 (2.19–9.92) (these incremental data is not shown in table). Overall, RMTAC + UC was found to be a dominant alternative compared to UC alone for both QALY gained and hospitalization averted outcomes.

**Table 3:** Outcomes and costs of base-case analysis

Outcome	RMTAC + UC	UC
Life years	27.88 (27.73–28.00)	27.76 (27.58–27.93)
QALYs	9.61 (7.97–11.29)	9.43 (7.85–11.00)
Number of hospitalization	16.60 (10.73–23.99)	22.22 (14.20–31.40)
Costs ($)*	16,370.35 (13,410.62–20,220.91)	18,780.21 (14,822.97–23,577.53)
ICER (vs. UC)		
$ per QALY gained	− 13,639.40 (− 109,556.90 to 104,445.54)	
$ per hospitalization averted	− 428.93 (− 521.27 to (− 328.69))	

All values are expressed in mean (95% credible intervals)

ICER, incremental cost-effectiveness ratio; QALY, quality-adjusted life year; RMTAC, Respiratory Medication Therapy Adherence Clinic; UC, usual care

* Cost is expressed in 2014 US dollars ($)

### One-way sensitivity analysis

**Fig. 3:**
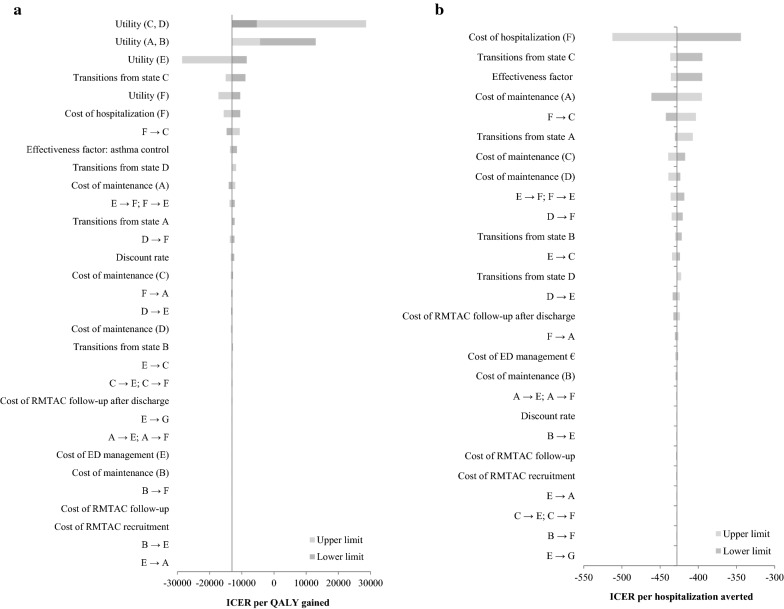
Results of one-way sensitivity analysis. The figures show the results of one-way sensitivity analysis for base-case ICER per QALY gained (**a**) and hospitalization averted (**B**) outcomes. The parameters involved correspond to those listed in Table 2. The width of the bars indicates the extent of variation of the base-case ICER due to change in one parameter at a time

The base-case results were found to be robust with all parameters varied within their range, except for the lower limit of utility of states A and B and the upper limit of utility of states C and D (Fig. 3a, b). In those two situations, RMTAC + UC was not considered as a cost-effective alternative compared to UC alone. These were expected as the lower limit of utility of states A and B was lower than the base-case utility of poor asthma control i.e. states C and D; hence the ICER increased to $12,955 per QALY gained. On the other hand, the upper limit of utility of states C and D was higher than the base-case utility of good asthma control i.e. states A and B; hence the ICER increased to $28,614 per QALY gained.

### Scenario analyses

The results of scenario analyses using PSA were found to be in a similar fashion as the base-case results (Fig. 2c–e). RMTAC + UC remained to be a dominant alternative compared to UC alone in both scenarios. The ICERs were higher than the ICER of base-case. At a lower effectiveness level, the mean QALY gained was 0.08 whilst a mean of 2 hospitalizations were averted. By contrast, at a lower set of utilities of exacerbations, the mean QALY gained was 0.30.

## Discussion

This study had shown that having pharmacist intervention (RMTAC) as an adjunct to clinical respiratory clinic (UC) was a cheaper and yet more effective alternative than UC alone in terms of QALY and hospitalizations.

Parameter inputs other than the transition probabilities between states A–D, utilities, and costs were from non-local sources due to lack of local data, hence there may be transferability issues. The two effectiveness factors originated from different countries i.e. Spain [21] and Australia [20]. Nevertheless, the characteristics of their interventions are deemed similar to RMTAC management because the pharmacists in those studies were trained prior to intervention, and conducted assessments and adjustments according to an individual needs on asthma control, medication adherence, and inhaler technique, gave education to patients regarding disease and medication issues; these tasks are almost, if not equally, identical to the RMTAC pharmacists’ tasks on every patient visit. Parameters obtained from observational studies that originated from United Kingdom (UK) and Spain were more readily transferable than US to Malaysia’s setting; data on mortality and exacerbations from UK and Spain have less impact on transferability issue because their extent of accessibility and affordability to healthcare services [40, 41] is similar to that in Malaysia. However, given that the accessibility and affordability in US is poor [40, 42], data on exacerbation and mortality rates from US observational studies are likely to be biased upwards in Malaysia setting; hence the ICERs in this study could be underestimated.

In this model, the same set of utilities was used for both arms. Although one might argue that RMTAC patients could have higher preferences for those health states than those who were not, similar studies have shown that there were no significant differences in the utilities between the intervention and control groups [43,44,45]. Therefore, data were collected from asthma patients regardless of their RMTAC treatment history.

The ICER per QALY gained in the base-case and scenario analyses seemed to be driven by the cost savings more than the QALY gained itself because of the small incremental of QALY between the two arms despite using two different sets of utilities. This could be explained by the insignificant difference between the good and poor asthma control utilities that were used as model inputs for states A–D. It is hypothesised that if the utility of poor asthma control is significantly lower than the utility of good asthma control, the incremental QALY will be larger. As such, the current ICERs could have been overestimated given the cost savings.

No direct comparison could be made with other literatures in terms of the base-case results, as there were no economic evaluations that assessed the efficiency of non-pharmacological asthma managements for a lifetime horizon [46]. Nevertheless, there was a 5-year study that demonstrated dominance of integrated care between respiratory nurse specialists, general physicians, and respiratory consultants [47]. Although the intervention is different than RMTAC, it still shows that asthma patients benefited from long-term enhanced management by gaining QALYs. No studies are known to have used hospitalization as outcome measure, however.

There are some limitations in this study. First, the monthly transition probabilities between states A–D were calculated from a small sample size (N = 68). Due to feasibility issue, data could not be collected from more than one public site to increase the sample size. As there were less than 20 observed monthly transitions, two methods were used to make the best out of all available observed transitions. Consequently, most of the probabilities had high uncertainties due to using non-monthly transitions to predict monthly transition probabilities. Despite this, the base-case ICER was found to be robust even when this parameter was varied within the upper and lower limits. Secondly, the modelled health states A, B, C, and D are made up of both asthma control and medication adherence elements. Ideally the treatment effect should be a composite of those elements. Due to lack of such data, individual effectiveness factor (asthma control) was used in the base case analysis instead and another (medication adherence) in the scenario analysis. The results from those analyses were similar; hence it seemed that there will not be a substantial difference in the ICER if a composite treatment effect even if it is necessary. Third limitation of the current study is that a Value-of Information analysis was not done to determine if the evidences used to build this model is sufficient and whether it is worth the money for further researches to be done given the uncertainties in the results, which ultimately affects the decision made by the health policymaker [48].

## Conclusions

Using a new lifetime Markov model that dynamically incorporates medication adherence, this study concludes that RMTAC + UC is a dominant alternative compared to UC alone from the MOH Malaysia perspective. For the benefit of both MOH and patients, RMTAC is thus recommended to be remained, and expanded to more healthcare settings where possible. It is suggested that data regarding effectiveness of RMTAC to be collected from more public healthcare settings in the future; the findings will be useful to address the uncertainties posed by the monthly transition probabilities between states A–D calculated here.

## Additional files

**Additional file 1: Appendix S1.** Calculation of transition probabilities

**Additional file 2: Appendix S2.** Calculation of transition probabilities for health states A – D of RMTAC + UC arm

## Abbreviations

CI: credible interval
ICER: incremental cost-effectiveness ratio
MOH: Ministry of Health
PSA: probabilistic sensitivity analysis
QALY: quality-adjusted life year
RMTAC: Respiratory Medication Therapy Adherence Clinic
UC: usual care
