# Supplementary material for: Using a dynamic adherence Markov model to assess the efficiency of Respiratory Medication Therapy Adherence Clinic (RMTAC) on asthma patients in Malaysia
Source: Cost Eff Resour Alloc. 2018 Oct 19;16:36. doi: 10.1186/s12962-018-0156-1 (PMC6195711; doi:10.1186/s12962-018-0156-1)
Supplement: Supplementary file 1 — Additional file 1: Appendix S1. Calculation of transition probabilities [file 12962_2018_156_MOESM1_ESM.docx]

APPENDIX S1

1. Transition probabilities for healthstates A – D of RMTAC + UC arm *(courtesy of Professor Henry Glick, University of Pennsylvania)*: Please refer to Appendix 2
2. Effectiveness factor
3. Asthma control Odds Ratio 3.059 (95% CI 1.632 - 5.733)

95% CI = 95% confidence interval

Convert to Relative Risk:

| RR = | OR |
| --- | --- |
|  | (1 – P_o_) + (P_o_ * OR) |

where P_o_ is the baseline prevalence or incidence in the non-exposed group

In this case, P_o_ is 42.7%

∴ RR = 3.059/((1-0.427)+(0.427*3.059)) = 1.628

Do the same for the 95% CI value:

∴ RR 1.628 (95% CI 1.285 – 1.898)

1. Medication adherence OR 1.89 (95% CI 1.08 - 3.30)

Convert to RR:

| RR = | OR |
| --- | --- |
|  | (1 – P_o_) + (P_o_ * OR) |

where P_o_ is the baseline prevalence or incidence in the non-exposed group

In this case, P_o_ is 60.3%

∴ RR = 1.89/((1-0.603)+(0.603*1.89)) = 1.230

Do the same for the 95% CI value:

∴ RR 1.230 (95% CI 1.030 – 1.383)

1. Transition probabilities for healthstates A – D of UC arm

Using the relative risk (RR) calculated in B, for transition to a worse state,

Transition probability for those states calculated in A * RR

Using the relative risk (RR) calculated in B, for transition to a better state,

Transition probability for those states calculated in A / RR

1. Monthly probabilities of low adherence level patient to have an exacerbation
2. B → E

From ref 23,

Number of good control patients had ED or OCS burst = 259

Number of person-years = 221.8

Hence, yearly rate of exacerbation = 259/221.8 = 1.168

Monthly rate of exacerbation = 1.168/12 = 0.097

Monthly probability of exacerbation =

*p* = 1 – e^-^*^rt^* where *r* is the rate and *t* is the duration of 1 month in this case

∴ Monthly probability of exacerbation = 0.093

1. B → F

From ref 23,

Number of good control patients had hospitalization due to asthma = 11

Number of person-years = 221.8

Hence, yearly rate of exacerbation = 11/221.8 = 0.049

Monthly rate of exacerbation = 0.049/12 = 0.004

Monthly probability of exacerbation =

*p* = 1 – e^-^*^rt^* where *r* is the rate and *t* is the duration of 1 month in this case

∴ Monthly probability of exacerbation = 0.004

1. D → E

From ref 23,

Number of poor control patients had ED or OCS burst = 3,346

Number of person-years = 1,054.4

Hence, yearly rate of exacerbation = 3,346/1,054.4 = 3.173

Monthly rate of exacerbation = 3.173/12 = 0.264

Monthly probability of exacerbation =

*p* = 1 – e^-^*^rt^* where *r* is the rate and *t* is the duration of 1 month in this case

∴ Monthly probability of exacerbation = 0.232

1. D → F

From ref 23,

Number of poor control patients had hospitalization due to asthma = 701

Number of person-years = 1054.5

Hence, yearly rate of exacerbation = 791/1054.5 = 0.665

Monthly rate of exacerbation = 0.665/12 = 0.055

Monthly probability of exacerbation =

*p* = 1 – e^-^*^rt^* where *r* is the rate and *t* is the duration of 1 month in this case

∴ Monthly probability of exacerbation = 0.054

1. Monthly probabilities of high adherence level patient to have an exacerbation
2. A → E

From ref 5,

Risk of A → E: HR 0.72 (95% CI 0.34 - 1.51) vs low adherence

*p* = HR / (1 + HR)

The contributional probability, *p* = 0.419 (in 6 months time)

Monthly rate, r = − (1/t)*ln(1 – p)

∴ r = 0.090

Monthly probability, *p* = 1 – e^-^*^rt^* where *r* is the rate and *t* is the duration of 1 month in this case

∴ Monthly contributional probability = 0.086

Probability of A → E = Multiply the monthly contributional probability to the probability of good control – low adherence (B → E = 0.093)

∴ 0.086*0.093 = 0.008

1. A → F

From ref 5,

Risk of A → F: HR 0.72 (95% CI 0.34 - 1.51) vs low adherence

*p* = HR / (1 + HR)

The contributional probability, *p* = 0.419 (in 6 months time)

Monthly rate, r = − (1/t)*ln(1 – p)

∴ r = 0.090

Monthly probability, *p* = 1 – e^-^*^rt^* where *r* is the rate and *t* is the duration of 1 month in this case

∴ Monthly contributional probability = 0.086

Probability of A → F = Multiply the monthly contributional probability to the probability of good control – low adherence (B → F = 0.004)

∴ 0.086*0.004 = 0.0003

1. C → E

From ref 5,

Risk of C → E: HR 0.59 (95% CI 0.37 - 0.95) vs low adherence

*p* = HR / (1 + HR)

The contributional probability, *p* = 0.371 (in 6 months time)

Monthly rate, r = − (1/t)*ln(1 – p)

∴ r = 0.077

Monthly probability, *p* = 1 – e^-^*^rt^* where *r* is the rate and *t* is the duration of 1 month in this case

∴ Monthly contributional probability = 0.074

Probability of C → E = Multiply the monthly contributional probability to the probability of good control – low adherence (D → E = 0.232)

∴ 0.074*0.232 = 0.017

1. C → F

From ref 5,

Risk of C → F: HR 0.59 (95% CI 0.37 - 0.95) vs low adherence

*p* = HR / (1 + HR)

The contributional probability, *p* = 0.371 (in 6 months time)

Monthly rate, r = − (1/t)*ln(1 – p)

∴ r = 0.077

Monthly probability, *p* = 1 – e^-^*^rt^* where *r* is the rate and *t* is the duration of 1 month in this case

∴ Monthly contributional probability = 0.074

Probability of C → F = Multiply the monthly contributional probability to the probability of good control – low adherence (D → F = 0.054)

∴ 0.074*0.054 = 0.004

1. Monthly probabilities of having good/poor asthma control after an exacerbation
2. E → A

From ref 25,

Number of patients previously had unscheduled visits and now having good asthma control = 251.2

Total number of patients previously had unscheduled visits in past a year= 949.5

Yearly probability = 251.2/949.5 = 0.265

Monthly rate, r = − (1/t)*ln(1 – p)

∴ r = 0.026

Monthly probability, *p* = 1 – e^-^*^rt^* where *r* is the rate and *t* is the duration of 1 month in this case

∴ Monthly probability = 0.025

1. E → C

From ref 25,

Number of patients previously had unscheduled visits and now having poor asthma control = 698.13

Total number of patients previously had unscheduled visits in past a year= 949.5

Yearly probability = 698.13/949.5 = 0.735

Monthly rate, r = − (1/t)*ln(1 – p)

∴ r = 0.111

Monthly probability, *p* = 1 – e^-^*^rt^* where *r* is the rate and *t* is the duration of 1 month in this case

∴ Monthly probability = 0.105

1. F → A

From ref 25,

Number of patients previously had hospitalizations and now having good asthma control = 34.1

Total number of patients previously had hospitalizations in past a year= 135

Yearly probability = 34.1/135 = 0.253

Monthly rate, r = − (1/t)*ln(1 – p)

∴ r = 0.024

Monthly probability, *p* = 1 – e^-^*^rt^* where *r* is the rate and *t* is the duration of 1 month in this case

∴ Monthly probability = 0.024

1. F → C

From ref 25,

Number of patients previously had hospitalizations and now having poor asthma control = 100.5

Total number of patients previously had hospitalizations in past a year= 135

Yearly probability = 100.5/135 = 0.7441

Monthly rate, r = − (1/t)*ln(1 – p)

∴ r = 0.114

Monthly probability, *p* = 1 – e^-^*^rt^* where *r* is the rate and *t* is the duration of 1 month in this case

∴ Monthly probability = 0.107

1. Monthly probabilities of having an exacerbation after a recent exacerbation

E → F and F → E

From ref 26

Proportion of patients having asthma-related re-admission within 30 days = 4.70%

∴ Monthly probability = 0.047

1. Monthly probability of mortality after an exacerbation that does not involve hospitalization

E → G

From ref 27

Number of death due to exacerbation but not hospitalization = 20 in 129 days

Total population = 79,233

Probability of death in 129 days = 0.00026

Daily rate, r = − (1/t)*ln(1 – p)

∴ r = 0.000001982

Monthly rate = 0.000001982*30 days

∴ r = 0.00005946

Monthly probability, *p* = 1 – e^-^*^rt^* where *r* is the rate and *t* is the duration of 1 month in this case

∴ Monthly probability = 0.000059458

1. Monthly probability of age-stratified mortality due to hospitalization

From ref 28,

The proportion of death in a year:

| Age (years) | Proportion of death (%) |
| --- | --- |
| 15-34 | 0.2 |
| 35-54 | 0.3 |
| 55-74 | 0.8 |
| 75 and above | 1.9 |

The probability of death in a year:

| Age (years) | Probability of death |
| --- | --- |
| 15-34 | 0.002 |
| 35-54 | 0.003 |
| 55-74 | 0.008 |
| 75 and above | 0.019 |

Using the formula of r = − (1/t)*ln(1 – p), the probability above was converted to annual rate

| Age (years) | Rate of death |
| --- | --- |
| 15-34 | 0.002002 |
| 35-54 | 0.003005 |
| 55-74 | 0.008032 |
| 75 and above | 0.019183 |

The annual rate was converted into monthly rate by dividing the rates with 12 months, and then converted to monthly probability using the formula of *p* = 1 – e^-^*^rt^*

| Age (years) | Monthly Probability |
| --- | --- |
| 15-34 | 0.000167 |
| 35-54 | 0.000250 |
| 55-74 | 0.000669 |
| 75 and above | 0.001597 |

1. Monthly probabilities of age-stratified mortality due to other causes

From ref 29,

The monthly probabilities were calculated using the same method as described above.
