# Supplementary material for: Using a dynamic adherence Markov model to assess the efficiency of Respiratory Medication Therapy Adherence Clinic (RMTAC) on asthma patients in Malaysia
Source: Cost Eff Resour Alloc. 2018 Oct 19;16:36. doi: 10.1186/s12962-018-0156-1 (PMC6195711; doi:10.1186/s12962-018-0156-1)
Supplement: Supplementary file 2 — Additional file 2: Appendix S2. Calculation of transition probabilities for health states A – D of RMTAC + UC arm [file 12962_2018_156_MOESM2_ESM.docx]

Henry Glick

08/10/15

**METHODS FOR CALCULATING TRANSITION PROBABILITIES**

**One-month observed transition probabilities**

Counts

|  | Time t+1 | | | | |
| --- | --- | --- | --- | --- | --- |
| Time t | A | B | C | D | Total |
| A | 1 | 0 | 1 | 0 | 2 |
| B | 1 | 1 | 0 | 0 | 2 |
| C | 2 | 1 | 4 | 1 | 8 |
| D | 2 | 0 | 0 | 2 | 4 |
| Total | 6 | 2 | 5 | 3 | 16 |

Transition probabililities

|  | Time t+1 | | | |
| --- | --- | --- | --- | --- |
| Time t | A | B | C | D |
| A | 0.50 | 0 | 0.50 | 0 |
| B | 0.50 | 0.50 | 0 | 0 |
| C | 0.25 | 0.125 | 0.5 | 0.125 |
| D | 0.5 | 0 | 0 | 0.5 |

Observations: As often as not, patients remain in the same state; When moving, they often move to either A or C

**Translating from “four-month” transition probabilities to one-month transition probabilities**

For this analysis, we classified three-, four-, and five-month transitions as a four-month transition (the decision rule for this analysis is to count any transition +/- one-month around the fourth month as a four-month transition). In the matrix below data are reported as three-month/four-month/five-month.

Counts (by months of follow-up)

|  | Time t+1 | | | | |
| --- | --- | --- | --- | --- | --- |
| Time t | A | B | C | D | Total |
| A | 0/3/4 | 2/1/0 | 0/3/0 | 1/0/0 | 3/7/4 |
| B | 2/1/2 | 0/2/1 | 0/0/0 | 0/0/0 | 2/3/3 |
| C | 1/4/1 | 0/0/0 | 5/8/0 | 0/0/1 | 6/12/2 |
| D | 0/0/0 | 0/0/1 | 2/0/1 | 3/3/0 | 5/3/2 |
| Total | 3/8/7 | 2/3/2 | 7/11/1 | 4/3/1 | 16/25 |

Summary Counts

|  | Time t+1 | | | | |
| --- | --- | --- | --- | --- | --- |
| Time t | A | B | C | D | Total |
| A | 7 | 3 | 3 | 1 | 14 |
| B | 5 | 3 | 0 | 0 | 8 |
| C | 6 | 0 | 13 | 1 | 20 |
| D | 0 | 1 | 3 | 6 | 10 |
| Total | 18 | 7 | 19 | 8 | 52 |

Four-month transition probabilities

|  | Time t+1 | | | |
| --- | --- | --- | --- | --- |
| Time t | A | B | C | D |
| A | 0.50 | 0.214 | 0.214 | 0.072 |
| B | 0.625 | 0.375 | 0 | 0 |
| C | 0.30 | 0 | 0.65 | 0.05 |
| D | 0 | 0.10 | 0.30 | 0.60 |

To obtain the square root of this matrix, we calculate its eigen vectors (a 4x4 matrix, V) and eigen values (a 1x4 matrix); we calculate the inverse of the eigen vector matrix (a 4 x 4 matrix, V^-1^); and we create a diagonal matrix made up of the square roots of the eigen values (D^½^, a 4x4 matrix with the principal diagonal made up of the square roots of the eigen values and the off-diagonal elements made up of 0s). The square root of the four-month transition matrix equals (V * D^½^ * V^-1^, where * represents matrix multiplication). (See “https://en.wikipedia.org/wiki/ Square_root_of_a_matrix”). The square root of the four-month transition matrix represents the predicted two-month transition matrix. We used the Stata “eigenstructure” command to calculate the eigen vector and eigen value matrices and the “luinv” command to calculate the inverse of the eigen vector matrix.

Four-month eigen vector matrix (V)

|  | Time t+1 | | | |
| --- | --- | --- | --- | --- |
| Time t |  |  |  |  |
|  | 0.5 | .167845246 | -.000944572 | -.445113892 |
|  | 0.5 | .488651077 | -.004720597 | .818851934 |
|  | 0.5 | -.133790911 | -.314636668 | .239355945 |
|  | 0.5 | -.845664289 | .949199974 | -.272146771 |

Inverse of four-month eigen vector matrix (V^-1^)

|  | Time t+1 | | | |
| --- | --- | --- | --- | --- |
| Time t |  |  |  |  |
|  | .789530198 | .306615631 | .677101098 | .226753073 |
|  | .901986604 | .713363034 | -1.21654118 | -.398808454 |
|  | .095317394 | .649518965 | -1.35314545 | .608309092 |
|  | -1.01980814 | .612043113 | .30472659 | .103038441 |

Four-month eigen value matrix

|  | Time t+1 | | | |
| --- | --- | --- | --- | --- |
| Time t | A | B | C | D |
|  | 1 | .589679315 | .500059984 | .0352607 |

Diagonal matrix (D^½^)

|  | Time t+1 | | | |
| --- | --- | --- | --- | --- |
| Time t |  |  |  |  |
|  | 1 | 0 | 0 | 0 |
|  | 0 | .767905799 | 0 | 0 |
|  | 0 | 0 | .707149195 | 0 |
|  | 0 | 0 | 0 | .187778327 |

m4msr = (V * D^½^ * V^-1^)

|  | Time t+1 | | | |
| --- | --- | --- | --- | --- |
| Time t | A | B | C | D |
| A | .596196248 | .193662615 | .157185316 | .052955821 |
| B | .57609816 | .512929897 | -.066569335 | -.022458722 |
| C | .23505226 | -.03698822 | .778300936 | .023635024 |
| D | -.074881404 | .094753244 | .204721921 | .775406239 |

As with the observed one-month transition matrix, the predicted two-month transition matrix generally has the largest probabilities for remaining in the state the patient began in. After that, the most frequent transitions are to state A. Of note, 4 of the predicted transition probabilities are negative.

Matrix multiplying m4msr times itself -- m4msr * m4msr (where * equals matrix multiplication) -- yields:

Four-month transition probabilities

|  | Time t+1 | | | |
| --- | --- | --- | --- | --- |
| Time t | A | B | C | D |
| A | 0.50 | 0.214 | 0.214 | 0.072 |
| B | 0.625 | 0.375 | 0 | 0 |
| C | 0.30 | 0 | 0.65 | 0.05 |
| D | 0 | 0.10 | 0.30 | 0.60 |

i.e., m4msr equals the square root of the four-month transition matrix.

To obtain predicted one-month transition probabilities from predicted two-month transition probabilities, we repeat the process but start with the predicted two-month transition probabilities.

Two-month eigen vector matrix (V)

|  | Time t+1 | | | |
| --- | --- | --- | --- | --- |
| Time t |  |  |  |  |
|  | 0.5 | -.167845246 | -.000944572 | -.445113892 |
|  | 0.5 | -.488651077 | -.004720597 | .818851934 |
|  | 0.5 | .133790911 | -.314636668 | .239355945 |
|  | 0.5 | .845664289 | .949199974 | -.272146771 |

Inverse of two-month eigen vector matrix (V^-1^)

|  | Time t+1 | | | |
| --- | --- | --- | --- | --- |
| Time t |  |  |  |  |
|  | .789530198 | .306615631 | .677101098 | .226753073 |
|  | -.901986604 | -.713363034 | 1.21654118 | .398808454 |
|  | .095317394 | .649518965 | -1.35314545 | .608309092 |
|  | -1.01980814 | .612043113 | .30472659 | .103038441 |

Two-month eigen value matrix

|  | Time t+1 | | | |
| --- | --- | --- | --- | --- |
| Time t | A | B | C | D |
|  | 1 | .767905799 | .707149195 | .187778327 |

Diagonal matrix (D^½^)

|  | Time t+1 | | | |
| --- | --- | --- | --- | --- |
| Time t |  |  |  |  |
|  | 1 | 0 | 0 | 0 |
|  | 0 | .876302344 | 0 | 0 |
|  | 0 | 0 | .840921634 | 0 |
|  | 0 | 0 | 0 | .433333967 |

m2msr = (V * D^½^ * V^-1^)

|  | Time t+1 | | | |
| --- | --- | --- | --- | --- |
| Time t | A | B | C | D |
| A | .72406007 | .139662908 | .101916049 | .034360973 |
| B | .418757868 | .673370992 | -.068880199 | -.023248661 |
| C | .158019912 | -.038698858 | .870807664 | .009871282 |
| D | -.077309895 | .070934057 | .124056618 | .88231922 |

As with the observed one-month transition matrix, the largest predicted transition probabilities are for remaining in the same state. There is very little movement from A to D or D to A (most movement between these two states requires prior movement to states B or C). Minimal movement is observed from B to C or D or from C to B or D.

Matrix multiplying m2msr itself four times – m2msr * m2msr * m2msr * m2msr (where * equals matrix multiplication) -- yields:

Four-month transition probabilities

|  | Time t+1 | | | |
| --- | --- | --- | --- | --- |
| Time t | A | B | C | D |
| A | 0.50 | 0.214 | 0.214 | 0.072 |
| B | 0.625 | 0.375 | 0 | 0 |
| C | 0.30 | 0 | 0.65 | 0.05 |
| D | 0 | 0.10 | 0.30 | 0.60 |

i.e., m2msr equals the fourth root of the four-month transition matrix.

**Combining observed and predicted one-month transition probabilities**

We have an observed one-month transition matrix based on 16 observations and a predited one-month transition matrix based on 52 observations.

One-month observed transition probabililities (N=16)

|  | Time t+1 | | | | Total Obs |
| --- | --- | --- | --- | --- | --- |
| Time t | A | B | C | D |  |
| A | 0.50 | 0 | 0.50 | 0 | 2 |
| B | 0.50 | 0.50 | 0 | 0 | 2 |
| C | 0.25 | 0.125 | 0.5 | 0.125 | 8 |
| D | 0.5 | 0 | 0 | 0.5 | 4 |

One-month predicted transition probabilities (N=52)

|  | Time t+1 | | | | Total Obs |
| --- | --- | --- | --- | --- | --- |
| Time t | A | B | C | D |  |
| A | .724 | .140 | .102 | .034 | 14 |
| B | .419 | .673 | -.069 | -.023 | 8 |
| C | .158 | -.039 | .871 | .010 | 20 |
| D | -.077 | .071 | .124 | .882 | 10 |

We combine the observed and predicted matrices by generating the weighted average of the row entries in the 2 matrices. For example, the weighted average for the transition from A to A is: ((0.5 * 2) + (0.724 * 14)) / 16 = 0.696; the weighted average for the transition from D to A is: ((0.5*4) + (-0.077 * 10)) / 14 = 0.088. The weighted average one-month transition probability matrix (with an effective sample size of 68 observations) equals:

|  | Time t+1 | | | | Total Obs |
| --- | --- | --- | --- | --- | --- |
| Time t | A | B | C | D |  |
| A | 0.696 | 0.122 | 0.152 | 0.030 | 16 |
| B | 0.435 | 0.638 | -0.055 | -0.018 | 10 |
| C | 0.184 | 0.008 | 0.765 | 0.043 | 28 |
| D | 0.088 | 0.051 | 0.088 | 0.773 | 14 |

The probability of a patient remaining in the same state as she began falls between the probabilities in the observed and predicted matrices, but still is the most frequent transition. Of the four transitions with negative probabilities in the one-month predicted matrix, two of the weighted averages are positive, while two remain negative.

**Addressing the two negative one-month transition probabilities**

Negative transition probabilities are predicted for transitions from B to C and B to D. Only 10 of the 68 one-, three-, four-, and five-month transitions were from State B, which makes B the state with the smallest number of patients from which transitions were made. None of the 10 transitions were from B to C or from B to D. Furthermore, none of the 11 observed two-month transitions started in state B. There is thus very little information that goes into the estimation of the negative probabilities and very little information for estimating what the actual probabilities should be. As a final step, by assumption, we zero-out the negative probabilities and adjust the probabilities of transitions from B to A and B to B proportionally so they sum to 1.0.

Revised B to A: 0.435 / (0.435+0.638) = 0.405

Revised B to B: 0.638 / (0.435+0.638) = 0.595

Revised B to C: 0

Revised B to D: 0

Thus, the final predicted one-year transition matrix equals:

|  | Time t+1 | | | | Total Obs |
| --- | --- | --- | --- | --- | --- |
| Time t | A | B | C | D |  |
| A | 0.696 | 0.122 | 0.152 | 0.030 | 16 |
| B | 0.405 | 0.595 | 0 | 0 | 10 |
| C | 0.184 | 0.008 | 0.765 | 0.043 | 28 |
| D | 0.088 | 0.051 | 0.088 | 0.773 | 14 |

**Two-month observed probabilities**

11 two-month transitions were observed in the dataset. When we tried to repeat this exercise, two of the eigen values were identical. As noted in the Wikipedia reference, the calculation requires that the eigen values be “distinct”. Implementation of the method describe above did not result in a square root matrix that when multiplied times itself resulted in the original two-month transition matrix. We thus did not use these 11 observations in our analysis.
